# Raltitrexed as a synergistic hyperthermia chemotherapy drug screened in patient-derived colorectal cancer organoids

**DOI:** 10.20892/j.issn.2095-3941.2020.0566

**Published:** 2021-08-15

**Authors:** Lisi Zeng, Quanxing Liao, Haoran Zhao, Shengwei Jiang, Xianzi Yang, Hongsheng Tang, Qingjun He, Xiansheng Yang, Shuxian Fang, Jinfu He, Weiwen Cui, Laiqiang Huang, Shaohua Ma, Shuzhong Cui

**Affiliations:** 1 Institute of Oncology, Affiliated Cancer Hospital and Institute of Guangzhou Medical University, Guangzhou 510095, China; 2 Department of Abdominal Surgery, Affiliated Cancer Hospital and Institute of Guangzhou Medical University, Guangzhou 510095, China; 3 Institute of Biopharmaceutical and Health Engineering, Shenzhen International Graduate School, Tsinghua University, Shenzhen 518055, China; 4 Tsinghua-Berkeley Shenzhen Institute (TBSI), Tsinghua University, Shenzhen 518055, China; 5 Shenzhen Key Laboratory of Gene and Antibody Therapy, Tsinghua University, Shenzhen 518055, China; 6 Department of Medical Oncology, Affiliated Cancer Hospital and Institute of Guangzhou Medical University, Guangzhou 510095, China; 7 Department of Bioengineering, University of California, Berkeley 94720, USA

**Keywords:** Colorectal cancer, organoids, hyperthermia chemotherapy sensitization enhancement ratio, raltitrexed

## Abstract

**Objective:**

Organoids have recently been used as *in vitro* models to screen chemotherapy drugs in combination with hyperthermia treatment in colorectal cancer. Our research aimed to establish a library of patient-derived colorectal cancer organoids to evaluate synergism between chemotherapy drugs and hyperthermia; validate an index of the hyperthermia chemotherapy sensitization enhancement ratio (HCSER) to identify the chemotherapeutics most enhanced by hyperthermia; and recommend chemotherapy drugs for hyperthermic intraperitoneal treatment.

**Methods:**

Organoids were grown from cells extracted from colorectal cancer patient samples or colorectal cancer cell lines. Cells from both sources were encapsulated in 3D Matrigel droplets, which were formulated in microfluidics and phase-transferred into identical cell-laden Matrigel microspheres. The microspheres were seeded in 96-well plates, with each well containing a single microsphere that developed into an organoid after 7 days. The organoids were used to evaluate the efficacy of chemotherapy drugs at both 37 °C as a control and 43 °C for 90 min to examine hyperthermia synergism. Cell viability was counted with 10% CCK8.

**Results:**

We successfully established a library of colorectal cancer organoids from 22 patient parental tumors. We examined the hyperthermia synergism of 7 commonly used hyperthermic intraperitoneal chemotherapy drugs. In 11 of the 22 patient organoids, raltitrexed had significant hyperthermia synergism, which was indexed as the highest HCSER score within each patient group.

**Conclusions:**

Our results primarily demonstrated the use of patient-derived colorectal cancer organoids as *in vitro* models to evaluate hyperthermia synergistic chemotherapeutics. We found that hyperthermia enhanced the effect of raltitrexed the most among the common anti-colorectal cancer drugs.

## Introduction

Colorectal cancer is the second most common cause of cancer deaths worldwide^1^, and the median life expectancy for metastatic colorectal cancer is only 16.3 months^2^. Among colorectal cancer metastases, peritoneal carcinomatosis (PC) is associated with particularly poor prognosis^3^. Hyperthermic intraperitoneal chemotherapy (HIPEC) was first introduced to treat PC in the 1980s^4^. Spratt and Sugarbaker^4,5^ then combined HIPEC with cytoreductive surgery, thus significantly improving prognosis in patients with metastatic colorectal cancer. HIPEC shows good treatment outcomes in PC caused by abdominal malignant tumors, such as colorectal cancer and ovarian cancer. In 2018, van Driel et al.^6^ demonstrated that, in patients with stage III epithelial ovarian cancer, cytoreductive surgery followed by HIPEC treatment significantly improves both recurrence-free and overall survival. HIPEC has become an accepted and commonly used adjunct in treating PC caused by colorectal cancer, gastric cancer, ovarian cancer, and other cancers^7^.

Hyperthermia is a method used to kill tumor cells by treating the tumor area or the whole body at an elevated temperature, usually between 39 °C and 48 °C, for one to several hours. Hyperthermia has a direct cytotoxic effect on tumor cells, as well as complementary and synergistic additive effects when combined with chemotherapy. However, no index is available to quantify the synergistic effects between individual drugs and hyperthermia.

Preclinical models that accurately reflect the biological characteristics of parental tumors are essential in evaluating and screening cancer therapeutics, particularly in personalized therapy^8,9^. Thus, colorectal cancer cell lines play a major role in colorectal cancer biology. However, these cell lines are limited because they lack the tumor microenvironment and have permanent gene alterations. Patient-derived xenografts solve the problem of the tumor microenvironment, but they require long times to establish, have low success rates, and are expensive. Meanwhile, whether mouse-based models can accurately simulate human diseases is a matter of debate. In this regard, the use of patient-derived organoids (PDOs) has led to the rapid development of precision cancer medicine^10–12^.

Organoids recapitulate the characteristics of tumors and their microenvironment in artificially engineered conditions, and they can overcome the shortcomings of cell lines in anticancer drug testing. They maintain homology with their primary tumors for as long as 1 year and more than 20 generations^13^. Organoid technology has developed into a high-throughput human biology analytic tool and is widely used to search for effective personalized treatment methods^11^. In the present study, we validated the application of PDOs in preclinical therapy screening for patient-variant colorectal cancer. We also identified raltitrexed as a chemotherapeutic drug that shows synergistic effects with hyperthermic intraperitoneal treatment.

## Materials and methods

### Human specimens and colorectal cancer cell lines

The present study was approved by the ethics committee of the Affiliated Cancer Hospital and Institute of Guangzhou Medical University. The colorectal cancer PDOs were derived from patients in the Affiliated Cancer Hospital and Institute of Guangzhou Medical University, between May and August 2020. The patients with colorectal cancer were diagnosed on the basis of pathology results, treated with cytoreductive surgery, and followed up with or without hyperthermic intraperitoneal chemotherapy. All colorectal cancer cell lines used in this study, including SW620, SW480, DLD-1, and COLO 205, were purchased from Procell Life Science & Technology Co. Ltd.

### Establishing colorectal cancer organoids

Colorectal cancer PDOs and colorectal cancer cell line organoids were harvested and dissociated into single cells according to the passaging procedure described below. The patient tumor specimens were immersed in an organic preservative solution (AQIX, UK) and transported from the hospital to the laboratory at 4 °C. The specimens were used to extract cells for fabricating colorectal cancer organoids.

Tumor tissues were washed 3 times in cold PBS (1×) solution containing 3.0% penicillin-streptomycin (v/v; Gibco). They were then cut into small pieces, and the tissues were digested with 1.0 mg/mL collagenase type I (Sigma-Aldrich), with 3.0% penicillin-streptomycin and 2.0% FBS (v/v, Invitrogen Tech), on an orbital shaker at 37 °C and for 1–2 h. After digestion, the tissues were sheared with 5 mL plastic pipettes. The suspension was passed through a 100 μm filter (Falcon) and centrifuged at 1,000 rpm for 5 min. The pellet was resuspended in Dulbecco’s modified Eagle’s medium/F12 medium. The number of extracted cells depended on the size of each tumor specimen—approximately 4.0 × 10^6^ cells were extracted from a fingernail-sized segment of tissue. The cells were counted with a hemocytometer.

The cells were then suspended in growth factor-reduced Matrigel (Corning) at a density of 2.0 × 10^7^ cells/mL. The Matrigel phase was subsequently loaded into a 1 mL syringe and installed in an injection pump. A fluorocarbon oil (HFE-7000; 3M Novec) was loaded into a 10 mL injection syringe and installed in another injection pump. Both pumps were placed in a refrigerator at 4 °C. These 2 phases were co-injected through polytetrafluoroethylene tubing (inner diameter, 600 μM; Woer) into a third piece of polytetrafluoroethylene tubing *via* a 3-way, handmade polydimethylsiloxane connector. The Matrigel phase was injected at a flow rate of 20 μL/min and subsequently sheared into monodisperse droplets by the fluorocarbon oil at a flow rate of 30 μL/min. The tubing carrying the Matrigel droplets, which was approximately 10 m long, was heated to 37 °C with a small water bath. The Matrigel droplets were circulated in the warmed tubing for 10 min before reaching the outlet, which was connected to the droplet printer head. The Matrigel beads were gelled from droplets and printed onto a 96-well plate. The printing sequences of the beads matched their sequences in the tubing. After printing, the beads were further incubated at 37 °C for 20 min to complete solidification. Next, 200 μL of culture medium was added to each well containing one cell-laden Matrigel bead, which constituted an organoid precursor. The culture medium consisted of 20% FBS, 1% penicillin-streptomycin, 100 ng/mL noggin (MCE), 100 ng/mL R-spondin 1 (MCE), 5 ng/mL epidermal growth factor (Peprotech), 10 ng/mL fibroblast growth factor-basic (MCE), 1× GlutaMAX supplement (Thermo Fisher Scientific), 10 mM HEPES (Thermo Fisher Scientific), 1× B-27 Supplement (Thermo Fisher Scientific), 5 mM nicotinamide (Sigma-Aldrich), 1.25 mM *N*-acetylcysteine (Sigma-Aldrich), 5 μM Y-27632 (Abmole), and 1× FibrOut™ (Chi Scientific). The preparation was then cultured at 37 °C in an incubator supplied with 5% CO_2_. The medium was changed every 3 days. Images of the organoids were acquired on days 1 and 7. The organoids were then harvested for further analysis or conditioned with drugs. Details on organoid fabrication and printing are described in another manuscript currently under review.

### Cell viability assay

Cell viability was assessed with Cell Counting Kit-8 (CCK-8) assays (Dojindo, Kumamoto, Japan). The cell suspension (100 μL) was dispensed into 96-well plates. CCK-8 solution (10 μL/well) was then added, and the preparation was incubated for 60 min. The absorbance was measured at 450 nm with a microplate reader. Cell viability was calculated as a percentage of the values for untreated controls. Half-maximal inhibitory concentration (IC_50_) values for each chemotherapy drug were calculated on the basis of cell viability curves in SPSS software (Version 26.0, USA).

### Drug screening by using colorectal cancer organoids

The colorectal cancer PDOs were established within 7 days. Cell line-derived organoids were established 3 days after the cell suspension was obtained. Subsequently, the culture medium was removed and replaced with 200 μL of drug-conditioned complete human organoid medium in each culturing well. The drug-conditioned medium was removed after 2 days and replaced with 100 μL of complete human organoid medium containing 10% CCK-8. The plates were placed back in the incubator (Thermo Fisher Scientific, Heraeus BB15). In most experiments, at least 3 replicates of each viability reading were acquired at each data point. Seven chemotherapy drugs were evaluated: oxaliplatin, lobaplatin, 5-fluorouracil, gemcitabine, mitomycin, raltitrexed, and abraxane. The concentration of each drug in the conditioned medium ranged from 0 to 1,000 μM (**Table 1**).

**Table 1:** Information on the chemodrugs used in this study

Drug name	Drug brand	Drug specifications
Raltitrexed	NANJING CHIA TAI TIANQING	2 mg
Mitomycin	HANHUI PHARMA	10 mg
Oxaliplatin	SANOFI	50 g
5-fluorouracil	PUDEP HARMA	0.25 g
Lobaplatin	Hainan Changan International Pharmaceutical Co. Ltd.	50 mg
Gemcitabine	NANJING CHIA TAI TIANQING	0.2 g
Abraxane	CSPC	100 mg

### Hyperthermia treatment of organoids

The tested organoids were seeded in 96-well plates and treated with hyperthermia. Specifically, the cells were heated in a cell incubator set at 43 °C for 90 min. They were then incubated at 37 °C in a regular cell culture incubator for another 48 h. Viability was determined with CCK-8 assays. The absorbance of the solution was measured at 450 nm, and the IC_50_, producing 50% cell death, was calculated *via* curve fitting in SPSS 26.0 software (Version 26.0, USA).

### Western blot

Total protein extracts were obtained from 6 colorectal cancer tissues with lysis buffer containing protease inhibitor. The protein concentrations were then determined with a bicinchoninic acid protein assay kit (KenGEN BioTECH, Nanjing, China). First, 30-μg protein samples were separated with SDS-PAGE and transferred to polyvinylidene fluoride membranes. The membranes were then blocked with 8% skimmed milk for 2 h at room temperature and incubated in antibody at 4 °C overnight. After being washed 3 times with TBST, the membranes were incubated with horseradish peroxidase-conjugated secondary antibodies at room temperature for 2 h. GAPDH was used as an internal control. Protein expression was visualized with a chemiluminescence system (Tanon 5200; Tanon Science & Technology Co. Ltd., Shanghai, China).

The information on primary antibodies and the respective applications in this study are shown in **Supplementary Table S1**.

### HIPEC procedure in patients

After surgical cytoreduction, HIPEC was administered for 90 min with chemotherapy drugs in 4–6 L of saline solution maintained at 43 °C and infused at a flow rate of 400–600 mL/min. The temperature of the perfusate was monitored with precision probes at multiple sites, including the entrance and exit ports, the tumor lesions, and the normal tissues. After perfusion was complete, the perfusate was drained. The fascia and skin were then closed according to standard surgical protocols.

### Statistical analysis

Statistical analysis was performed in SPSS 26.0 and GraphPad Prism 7.0 software (GraphPad Software Inc., La Jolla, CA, USA). IC_50_ calculations were obtained with 4–6 concentrations of each compound and generated in SPSS 26.0 Logit. All data are expressed as mean ± standard deviation. Student’s unpaired t-test were used to compare continuous variables between two groups, whereas Pearson’s χ^2^ test or Fisher’s exact test were used to compare categorical variables between two groups. *P* < 0.05 indicated a statistically significant difference.

## Results

### Establishment of PDOs by using *in vitro* tumor models of colorectal cancer

**Figure 1:**
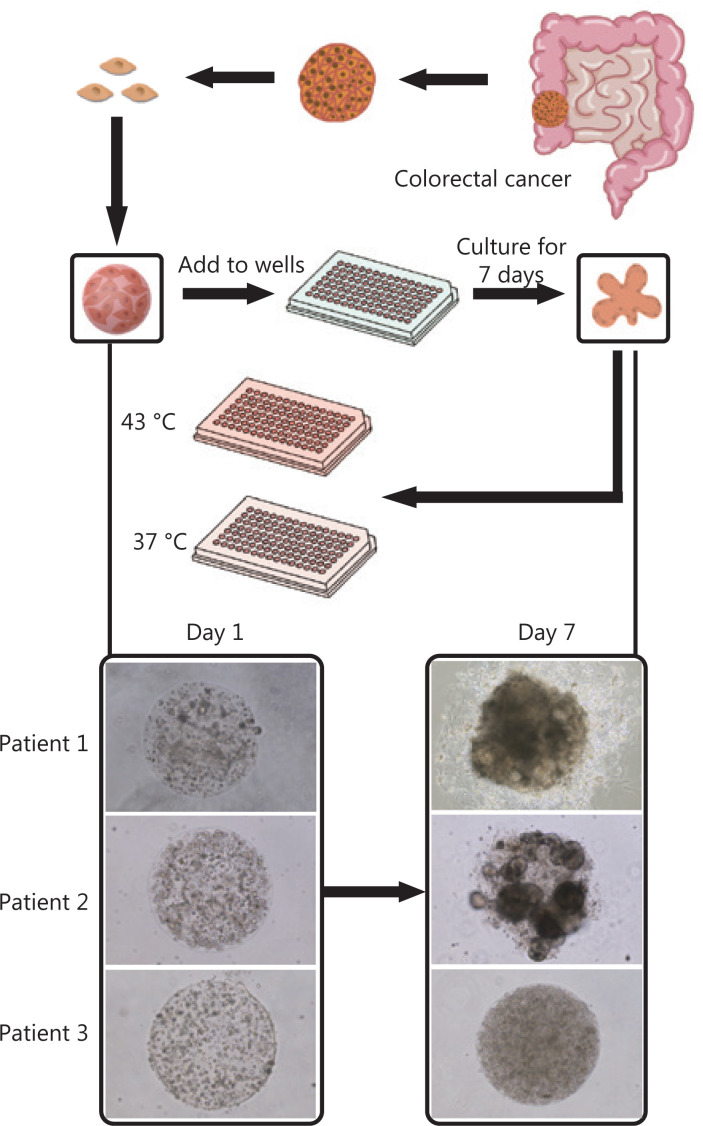
Establishment of patient-derived organoids as *in vitro* tumor models for colorectal cancer. Morphology of patient-derived organoids (PDOs) on days 1 and 7. After 7-day culture, the organoids were used to evaluate hyperthermia and chemotherapy. The figure shows a flowchart of hyperthermia and chemotherapy evaluation. PDOs are bifurcated to evaluate the following chemotherapy drugs at 37 °C and 43 °C: mitomycin, abraxane, 5-fluorouracil, oxaliplatin, raltitrexed, gemcitabine, and lobaplatin.

The colorectal cancer organoids were derived from the surgical specimens of 22 patients. Colorectal cancer cell lines, including SW620, SW480, DLD-1, and COLO 205, were also used to construct organoids for parallel studies. The cells from both sources were encapsulated into monodisperse, cell-laden Matrigel tumor microspheres (diameter, 600 μm) and then cultured as suspensions in medium in 96-well plates. Cell line-derived organoids or PDOs were obtained after cell proliferation and self-organization in the individual microspheres for 3 days (for cell lines) or 1 week (for PDOs) (**Figure 1**). According to the 2019 version of the consensus reached by Chinese medical experts on the clinical applications of HIPEC^7^, the commonly used HIPEC drugs for colorectal cancer include mitomycin, oxaliplatin, and 5-fluorouracil. Other chemotherapy drugs are on the recommended list but require further exploration. Notably, the recommended concentrations of chemotherapy drugs in HIPEC are usually 1–2 orders of magnitude higher than those used in intravenous chemotherapy. On this basis, we designed an investigation portfolio comprising these 3 common drugs and 4 other pending drugs, with dose variations. The median doses were arbitrarily chosen; however, they were generally 1–2 orders of magnitude higher than the commonly responsive doses reported for organoid screening (**Table 2**). The organoids were conditioned with single drugs at the concentrations listed in **Table 2** at 37 °C and 43 °C for 90 min.

**Table 2:** Drug doses used clinically and in experiments

Drug name	Recommended dose in clinically of single chemotherapy	Intraperitoneal perfusion concentration	Experimental setting
Raltitrexed	3 mg/m^2^	2.8 μM	0/0.2/2/20/200/2,000 μM
Mitomycin	6–8 mg	5.9 μM	0/0.05/0.5/5/50/500 μM
Oxaliplatin	130	139 μM	0/0.1/1/10/100/1,000 μM
5-Fu	500–600 mg/m^2^	1.96 μM	0/0.02/0.2/2/20/200 μM
Lobaplatin	50 mg/m^2^	53.5 μM	0/0.05/0.5/5/50/500 μM
Gemcitabine	1,000/1,250 mg/m^2^	1.8 μM	0/0.02/0.2/2/200/2,000 μM
Abraxane	260 mg/m^2^	122.9 μM	0/0.1/1/10/100/1,000 μM

### Raltitrexed has the highest hyperthermia chemotherapy sensitization enhancement ratio score among the screened chemotherapy drugs

To assess whether our colorectal cancer organoids were applicable as *in vitro* tumor models to evaluate patient-dependent variance in chemotherapy drug sensitivity, we generated dose-response curves for the 7 drugs and calculated their half-maximal inhibitory concentrations (IC_50_). The drug library was composed of oxaliplatin, lobaplatin, 5-fluorouracil, gemcitabine, mitomycin, raltitrexed, and abraxane. The hyperthermia chemotherapy sensitization enhancement ratio (HCSER) was used to evaluate the sensitizing effect of hyperthermia on chemotherapy. The HCSER is the ratio of the concentration of a drug applied to the conditions of chemotherapy alone or in combination with hyperthermia therapy that results in the same consequence of killing tumor cells, for example, 50% residual viability. The formula was HCSER = IC_50_ (37 °C)/IC_50_ (43 °C). In 11 of 22 patient organoids, raltitrexed had the highest HCSER score within each patient group. The differences in the hyperthermia chemotherapy sensitization enhancement ratio were statistically significant (*P* < 0.01) between the high-HCSER group and the low-HCSER group (**Table 3**). All IC_50_ values, HCSER scores, and their standard deviations are provided in **Supplementary Table S2**.

**Table 3:** Comparison of HCSER between the high-HCSER group and the low-HCSER group

High-HCSER group			Low-HCSER group		
Cases	Average	SD	Cases	Average	SD
11	167.37	297.4	11	4.89	5.90
*P*	0.0019				

The High-HCSER group contains patients 1–11, and the low-HCSER group contains patients 12–22. SD, standard deviation. *P* < 0.05 was considered statistically significant.

Raltitrexed is an anti-metabolic folate analog that specifically inhibits thymidylate synthase (TS)^14,15^, a key enzyme in the synthesis of thymidine triphosphate, a nucleotide necessary for DNA synthesis. Inhibition of TS can lead to DNA breakage and apoptosis^16^. Raltitrexed is a direct and specific TS inhibitor that can enhance the inhibition ability of TS and prolong the inhibition time^17^.

**Figure 2:**
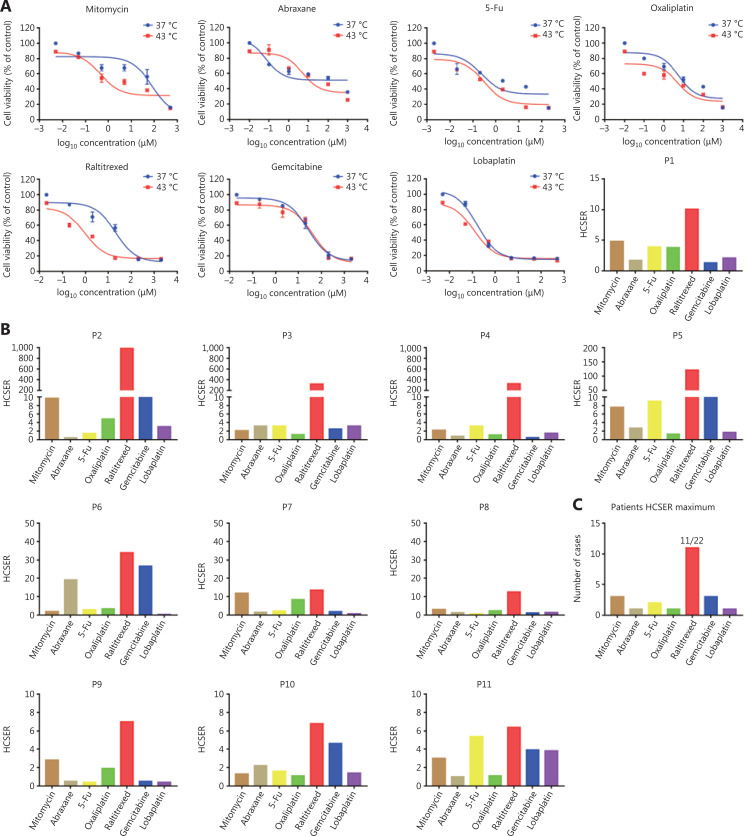

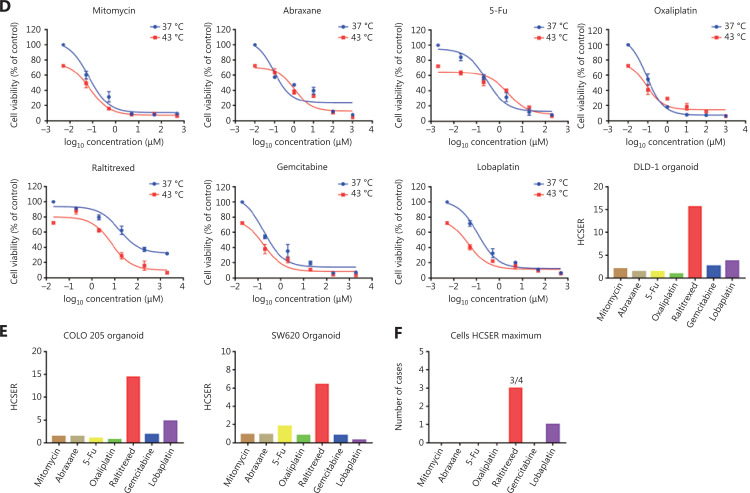
Raltitrexed has the highest hyperthermia chemotherapy sensitization enhancement ratio score among the screened chemotherapy drugs. (A) The dose-response curves of organoids derived from patient 1 after treatment with different drugs for 90 min at 37 °C and 43 °C. Cell viability at 37 °C was measured with 10% CCK-8 after 2 days under drug-free conditions after drug treatment. The hyperthermia chemotherapy sensitization enhancement ratio (HCSER) score was obtained by calculating the ratio of the IC_50_ values between 37 °C and 43 °C for each drug. That is, HCSER = IC_50_ (37 °C)/IC_50_ (43 °C). (B) The HCSER scores of organoids derived from the other 10 patients. Raltitrexed had the highest HCSER score. (C) The highest HCSER scores of the 7 chemotherapy drugs in organoids from 22 patients. (D) Dose-response curves of organoids derived from DLD-1 cell lines, and the HCSER summary for all evaluated drugs. (E) The HCSER scores of the organoids derived from another 2 colorectal cancer cell lines. Raltitrexed showed the highest HCSER scores. (F) Counts of the highest HCSER scores of the 7 chemotherapy drugs in the 4 colorectal cancer cell lines.

Organoids derived from patient 1 were tested with the 7 single drugs at both 37 °C and 43 °C in parallel. Raltitrexed had the highest HCSER of 10.1 (**Figure 2A**). Among the 22 patients investigated, raltitrexed had the highest HCSER score under hyperthermia synergism in 11 patients (**Figure 2B and 2C**). In the other 11 patients, the drugs with the highest HCSER scores obtained from the PDO library were mitomycin (3 of 22 patients), gemcitabine (3 of 22 patients), 5-Fu (2 of 22 patients), abraxane (1 of 22 patients), oxaliplatin (1 of 22 patients), and lobaplatin (1 of 22 patients) (**Supplementary Figure S1**).

We performed a similar evaluation with colorectal cancer cell lines. Raltitrexed showed the highest HCSER scores in most of the cell lines investigated, including DLD-1, SW620, and COLO 205, although lobaplatin had the highest HCSER score in the SW480 cell line (**Figures 2D–2F and Supplementary S2**). These findings suggest that raltitrexed has a high level of hyperthermia synergism in the treatment of colorectal cancers, and that this drug should be included on the recommendation list for clinical administration of HIPEC.

### Effects of raltitrexed on biological processes in colorectal cancer

Next, to ascertain how raltitrexed confers hyperthermia sensitization, we performed transcriptomic analysis with RNA sequencing (RNA-seq) of 5 primary tumors that were distinctively sensitive or non-sensitive to raltitrexed. The analysis included tumors from 3 patients (P15, P20, and P22) who were insensitive to raltitrexed (HCSER < 2) and 2 patients (P5 and P10) who were highly sensitive (HCSER > 6). More than 1,200 differentially expressed genes were identified between the raltitrexed sensitive and insensitive groups, with screening criteria of log_2_ (fold change) > 0.5 or < −0.5, and a *P*-value ≤ 0.05.

**Figure 3:**
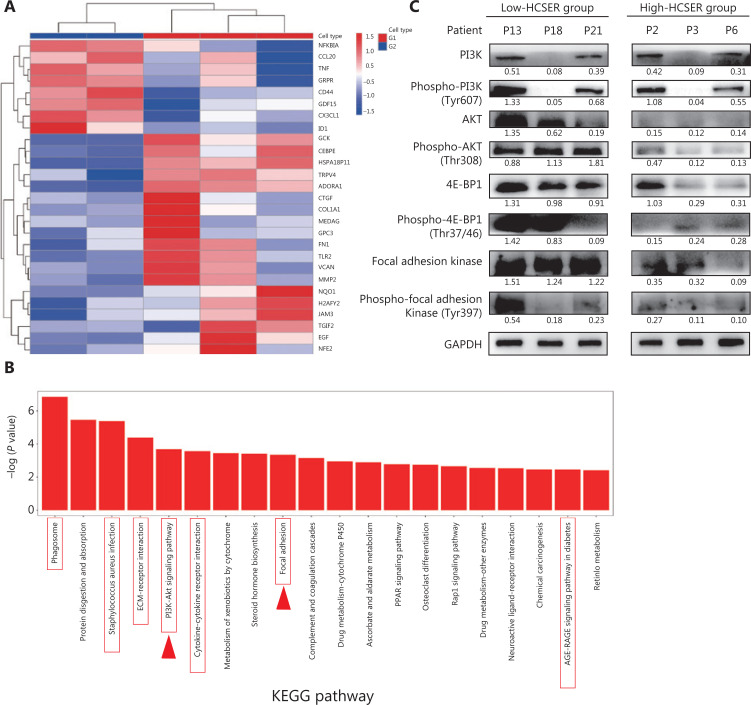
Gene expression profiles of raltitrexed hyperthermia sensitization. (A) Clustered heat map of differentially expressed genes. G1 denotes patients insensitive to raltitrexed (P15, P20, and P22). G2 denotes patients sensitive to raltitrexed (P5 and P10). (B) Enrichment in KEGG pathway terms for differentially expressed genes between the G1 and G2 groups. The horizontal axis denotes the enriched KEGG pathways, and the vertical axis displays the −log_10_ (*P*-value). The red boxes represent the 27 genes participating in these pathways, and the red arrows represent pathways reported to be associated with hyperthermia. (C) Western blot analysis was conducted to assay the downregulated expression of PI3K, p-PI3K, AKT, p-AKT, 4E-BP1, p-4E-BP1, Focal Adhesion Kinase, and p-Focal Adhesion Kinase in the raltitrexed high-HCSER group. The low-HCSER group contains patients 13, 18, and 21, and the high-HCSER group contains patients 2, 3, and 6.

Twenty-seven genes showed a synergistic correlation between hyperthermia and chemotherapy (**Table 4**). According to the differentially expressed genes screened among the sampled groups, the genes and samples were clustered bidirectionally and displayed on a heat map (**Figure 3A**). We performed KEGG pathway analysis, which showed that numerous pathways might contribute to the differential sensitivity, including PI3K-AKT signaling^18^ and focal adhesion^19^ pathways that affect hyperthermia (**Figure 3B**). To investigate whether hyperthermia enhanced raltitrexed treatment by regulating the PI3K/AKT/mTOR and Focal Adhesion Kinase signaling pathways, we performed Western blot on tumors from 3 patients (P13, P18, and P21) with high HCSER scores and another 3 patients (P2, P3, and P6) with low HCSER scores. The expression levels of PI3K, p-PI3K, AKT, p-AKT, 4E-BP1, p-4E-BP1, Focal Adhesion Kinase, and p-Focal Adhesion Kinase were downregulated in the high-HCSER group. These findings suggested that hyperthermia may enhance raltitrexed in colorectal cancer by inhibiting the PI3K/AKT/mTOR and Focal Adhesion Kinase signaling pathways (**Figure 3C**).

**Table 4:** Information on 27 genes previously reported to be associated with hyperthermia

Gene	Title of related article	PMID
NFKBIA	Short-term hyperthermia prevents activation of proinflammatory genes in fibroblast-like synoviocytes by blocking the activation of the transcription factor NF-kappaB.	16955275
CCL20	Local hyperthermia decreases the expression of CCL-20 in condyloma acuminatum.	21050487
TNF	Comparative *in vitro* studies of the potentiation of tumor necrosis factor (TNF)-alpha, TNF-beta, and TNF-SAM2 cytotoxicity by hyperthermia.	1571335
GRPR	How gastrin-releasing peptide receptor (GRPR) and α(v)β(3) integrin expression reflect reorganization features of tumors after hyperthermia treatments.	28761146
CD44	CD44-targeted magnetic nanoparticles kill head and neck squamous cell carcinoma stem cells in an alternating magnetic field.	31571863
GDF15	Involvement of ERK1/2 signalling and growth-related molecules’ expression in response to heat stress-induced damage in rat jejunum and IEC-6 cells.	20707649
CX3CL1	Heat therapy promotes the expression of angiogenic regulators in human skeletal muscle.	27357800
ID1	Correlation between the expression of Id-1 and hyperthermia-associated molecules in oral squamous cell carcinoma.	23723304
GCK	Cellular signalling after *in vivo* heat shock in the liver.	10772775
CEBPE	Gene networks involved in apoptosis induced by hyperthermia in human lymphoma U937 cells.	19732844
HSPA8P11	The cellular and molecular basis of hyperthermia.	12098606
TRPV4	Ischemic brain injury leads to brain edema *via* hyperthermia-induced TRPV4 activation.	29793978
ADORA1	Activation of central adenosine A(2A) receptors lowers the seizure threshold of hyperthermia-induced seizure in childhood rats.	21144776
CTGF	Role of CTGF in sensitivity to hyperthermia in ovarian and uterine cancers.	27806300
COL1A1	Molecular pathology of vertebral deformities in hyperthermic Atlantic salmon (Salmo salar).	20604915
MEDAG	Hyperthermia severely affects the vascular effects of MDMA and metabolites in the human internal mammary artery* in vitro*.	28084566
GPC3	Glypican-3 (GPC3) targeted Fe_3_O_4_ core/Au shell nanocomplex for fluorescence/MRI/photoacoustic imaging-guided tumor photothermal therapy.	31603456
FN1	Role of CTGF in sensitivity to hyperthermia in ovarian and uterine cancers.	27806300
TLR2	Extracellular heat shock protein 70 mediates heat stress-induced epidermal growth factor receptor transactivation in A431 carcinoma cells.	17126326
VCAN	Versican and vascular endothelial growth factor expression levels in peritoneal metastases from colorectal cancer are associated with survival after cytoreductive surgery and hyperthermic intraperitoneal chemotherapy.	26873137
MMP2	Hyperthermia inhibits the motility of gemcitabine-resistant pancreatic cancer PANC-1 cells through the inhibition of epithelial-mesenchymal transition.	29568909
NQO1	Anti-cancer effect of bio-reductive drug beta-lapachon is enhanced by activating NQO1 with heat shock.	18283592
H2AFY2	Proteomic and bioinformatic analysis of condyloma acuminata: mild hyperthermia treatment reveals compromised HPV infectivity of keratinocytes *via* regulation of metabolism, differentiation and anti-viral responses.	30909744
JAM3	Core temperature correlates with expression of selected stress and immunomodulatory genes in febrile patients with sepsis and noninfectious SIRS.	19496026
TGIF2	Involvement of ERK1/2 signalling and growth-related molecules’ expression in response to heat stress-induced damage in rat jejunum and IEC-6 cells.	20707649
EGF	Effect of hyperthermia on invasion ability and TGF-β1 expression of breast carcinoma MCF-7 cells.	21455587
NFE2	Hyperthermia and protein homeostasis: cytoprotection and cell death.	32716865

### Colorectal cancer PDOs reflect the clinical outcomes of representative patients with colorectal cancer

We collected surgically removed colorectal cancer tissues from 22 patients (**Table 5**), all of whom had undergone radical colorectal cancer resection, and 6 of whom had received HIPEC treatment after surgery. The drugs for HIPEC included raltitrexed, mitomycin, and oxaliplatin.

**Table 5:** Clinical and pathological features of colorectal cancer patients in 2 groups

Characteristics	Surgery (*n* = 16)	Surgery + HIPEC (*n* = 6)	Total (*n* = 22)
Age			
Mean ± SD	58.1 *±* 13.5	54.2 *±* 3.5	57.0 *±* 11.7
Range	37–82	50–60	37–82
Gender			
Male	10	3	13
Female	6	3	9
Primary tumor site			
Ascending colon	3	1	4
Transverse colon	1	0	1
Descending colon	1	0	1
Sigmoid colon	4	3	7
Rectum	7	2	9
Drug used in HIPEC			
Mitomycin	0	3	3
Raltitrexed	0	1	1
Raltitrexed + mitomycin + oxaliplatin	0	2	2
Surgical modality			
Radical surgery	14	3	17
Palliative surgery	2	3	5
TNM stage			
Stage I	2	1	3
Stage II	4	2	6
Stage III	8	0	8
Stage IV	2	3	5
Metastatic sites			
Liver	2	2	4
Adrenal gland	1	0	1
Abdominal cavity	0	1	1
CEA (normal: < 5.0 ng/mL)			
Normal	9	3	12
Abnormal	7	3	10
CA19-9 (normal: < 30 U/mL)			
Normal	13	4	17
Abnormal	3	2	5
CA72-4 (normal: < 6.9 U/mL)			
Normal	11	4	15
Abnormal	5	2	7

**Figure 4:**
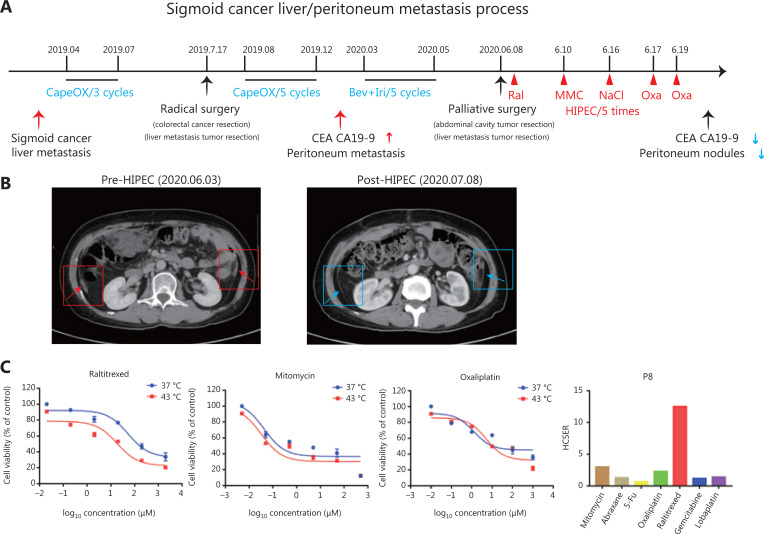
Patient-derived organoids reflect personal response to chemotherapy in combination with hyperthermic intraperitoneal chemotherapy. (A) Treatment and procedure timeline in patient 8. The values highlighted in blue indicate the administration of chemotherapy. The values highlighted in red indicate the efficacy of hyperthermic intraperitoneal chemotherapy. (B) Left: CT image of patient 8 before surgery. Right: CT image of patient 8 at 1 month after surgery. The small nodules and peritoneal fluid decreased, as compared with those in the left CT image. (C) Cell viability of organoids derived from patient 8 in response to raltitrexed, mitomycin, and oxaliplatin, and the HCSER for those drugs.

Patient 8 was diagnosed with sigmoid cancer combined with live metastasis in April 2019. She received 3 rounds of CapeOX (capecitabine and oxaliplatin) to reduce the tumor size in preparation for surgery. On July 17, 2019, the patient underwent colorectal cancer resection and liver metastasis tumor resection surgery, followed by 5 rounds of CapeOX (**Figure 4A**). However, 7 months after surgery, she experienced peritoneal metastasis. Computed tomography (CT) showed peritoneal thickening, and the expression of tumor markers (CEA and CA19-9) increased. After 5 rounds of chemotherapy, her overall condition had not improved. On June 6, 2020, she underwent palliative resection of an abdominal cavity tumor and a liver metastatic tumor. Liver metastases and metastasis nodules were found in the omentum and abdominal wall.

Small intraperitoneal metastatic lesions were difficult to remove by surgery. Therefore, after the palliative resection surgery, Patient 8 received 5 rounds of HIPEC treatment with raltitrexed, mitomycin, and oxaliplatin on day 1 (24 h), day 2, day 8, day 9, and day 11. The second HIPEC therapy did not use any drug, but only 0.9% NaCl. At 1 month after surgery, CT revealed small nodules and reduced peritoneal fluid, thus indicating that HIPEC had relieved the symptoms in this patient (**Figure 4B**). The drug responses of the organoids derived from the same patient showed that raltitrexed, mitomycin, and oxaliplatin had high HCSER scores, thereby validating our PDOs for screening personalized chemotherapy drugs in HIPEC therapies (**Figure 4C**). These results also indicated that hyperthermia enhanced the effect of raltitrexed the most among the common anti-colorectal cancer drugs.

## Discussion

A worldwide consensus on chemotherapy drugs in HIPEC currently exists^20^. Mitomycin and oxaliplatin are the most frequently used drugs in clinical and research protocols. However, because of the absence of effective pre-clinical models to evaluate drug efficacy, the performance and clinical recommendations for these drugs remain under debate^21^. Moreover, other clinically approved drugs should be added to HIPEC regimens to expand the selection pool and allow surgeons to match inter-patient variations in drug sensitivity. Hence, in the present study, we introduced PDOs as pre-clinical tumor models to evaluate HIPEC-associated chemotherapy drugs. We validated the reliability of PDOs for this purpose in a small library of samples from patients with colorectal cancer. In conjunction with PDO evaluation, we introduced the HCSER to evaluate HIPEC-associated drug choice. The HCSER was calculated with the IC_50_ of a drug at 37 °C and 43 °C treatment. In this way, it was used to examine the hyperthermia sensitization of chemotherapy drugs.

We found that raltitrexed had the highest HCSER score among the screened drugs, thus implying that it should be included in the clinical recommendations for HIPEC treatment. However, further pre-clinical studies should be conducted before such action is taken, because the current study has some limitations. For example, the patient cohort was fairly small. To further validate our findings, an expanded library with a richer diversity of samples should be established. For example, patients should be recruited from different regions of China or even worldwide. The performance of this treatment regimen in other cancers should also be studied to draw conclusions beyond the scope of colorectal cancer.

Further mechanistic study of hyperthermia sensitization is required for therapeutic improvement. We performed a sequence study on 2 samples that were sensitive to raltitrexed and 3 that were insensitive. The RNA sequencing results showed that 8 hyperthermia-related genes were upregulated in the raltitrexed-sensitive group, and 19 hyperthermia- related genes were downregulated. KEGG analysis showed that the PI3K-AKT and focal adhesion signaling pathways might be involved in the hyperthermia sensitization to raltitrexed. Huang et al.^19^ have reported that hyperthermia may disrupt the integrin-mediated actin cytoskeleton in thyroid carcinoma by affecting focal adhesions. Fang et al.^18^ have confirmed that hyperthermia improves the sensitivity of Raji cells to chemotherapy drugs by blocking the PI3K/AKT pathway and downregulating HSP70. However, further studies must be performed to ascertain the mechanism.

Personalized precision treatment of cancer has become a focus of medical research and clinical practice worldwide. PDOs have been suggested as personalized cancer screening models that can reflect inter-patient variability in drug sensitivity^22–25^. Furthermore, they are promising in the evaluation of personalized synergism between hyperthermia and chemotherapy. The present study indicated that PDOs are beneficial in evaluating personalized chemotherapy drug selection in HIPEC therapy. Additional clinical validation will be performed in follow-up studies.

## Conclusions

In brief, our research primarily demonstrated that colorectal cancer PDOs can be used as *in vitro* cancer models to evaluate hyperthermia synergistic chemotherapeutics. We found that hyperthermia enhanced the effect of raltitrexed the most among the common anti-colorectal cancer drugs.
