# Supplementary material for: Raltitrexed as a synergistic hyperthermia chemotherapy drug screened in patient-derived colorectal cancer organoids
Source: Cancer Biol Med. 2021 Aug 15;18(3):750–62. doi: 10.20892/j.issn.2095-3941.2020.0566 (PMC8330527; doi:10.20892/j.issn.2095-3941.2020.0566)
Supplement: Supplementary file 1 [file cbm-18-750-s001.pdf]

Supplementary materials

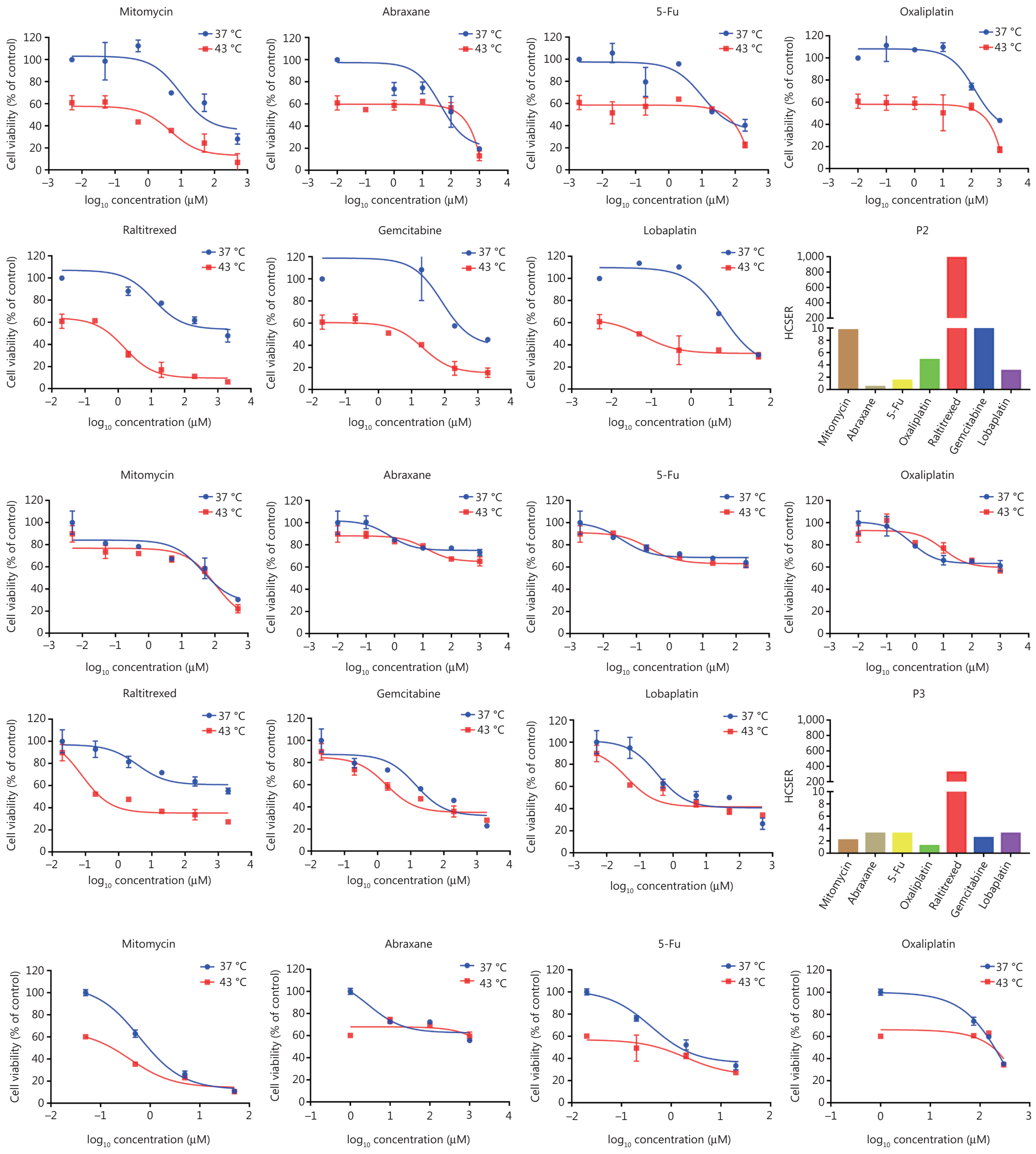

Figure S1 Continued

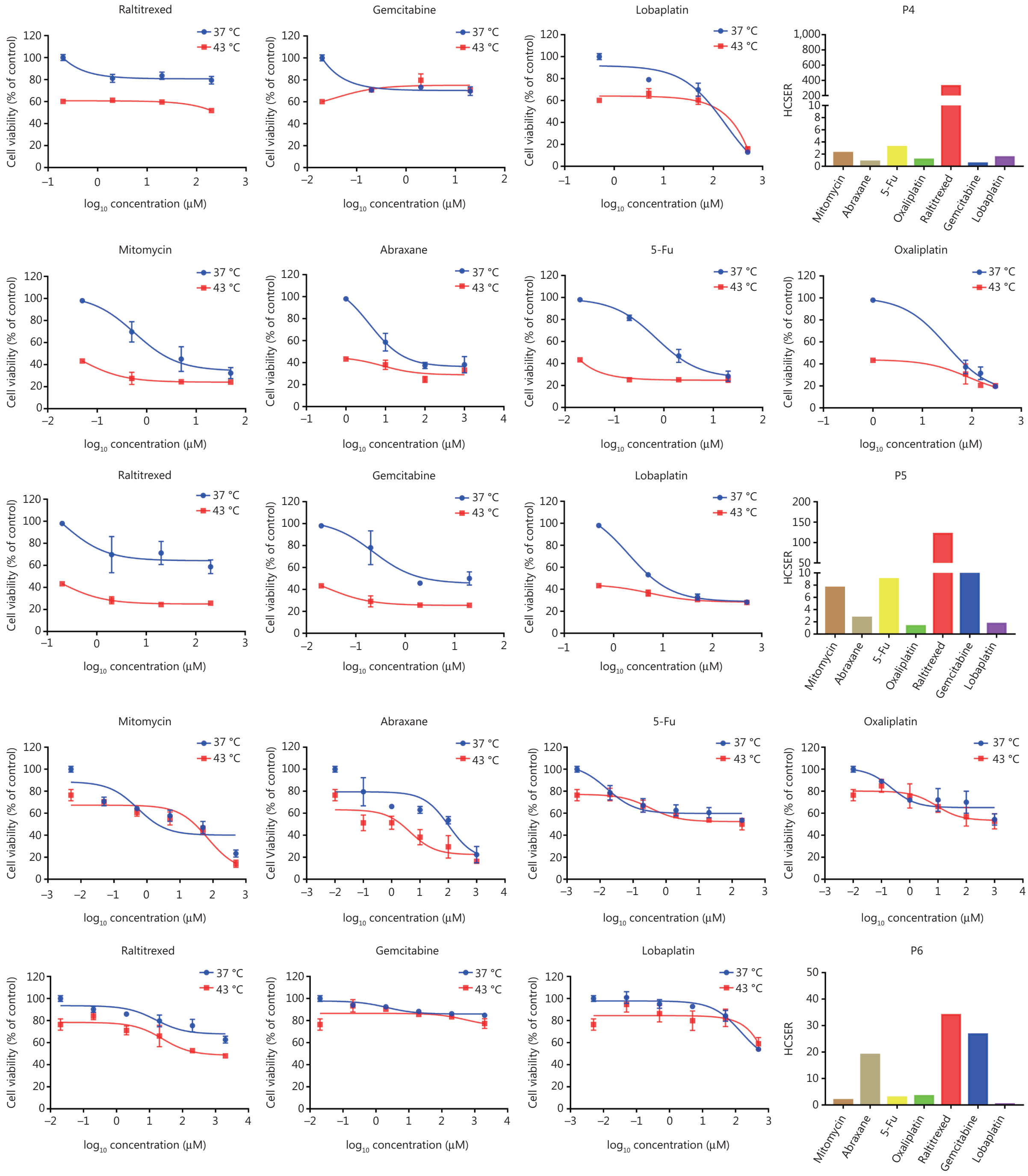

Figure S1 Continued

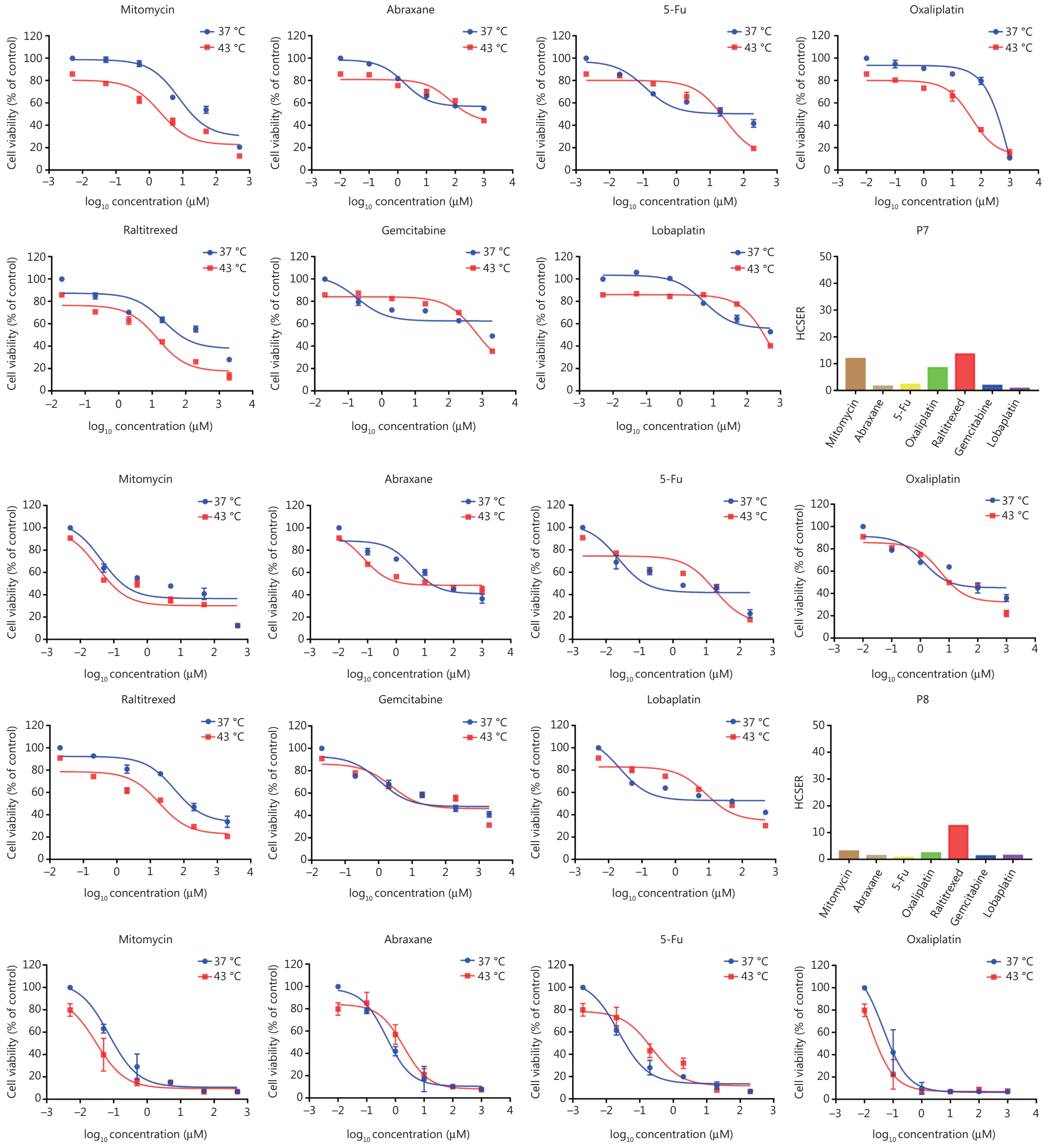

Figure S1 Continued

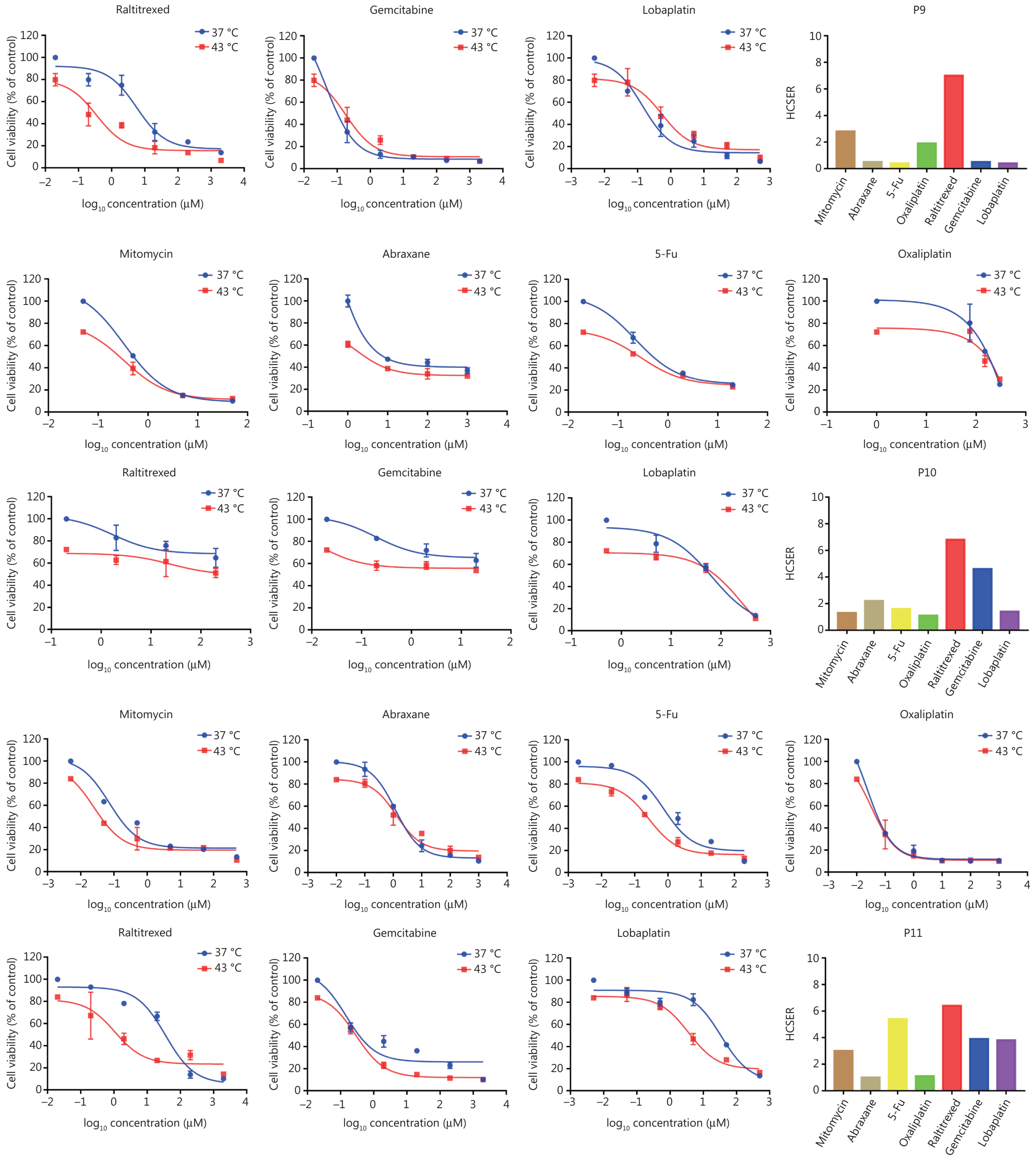

Figure S1 Continued

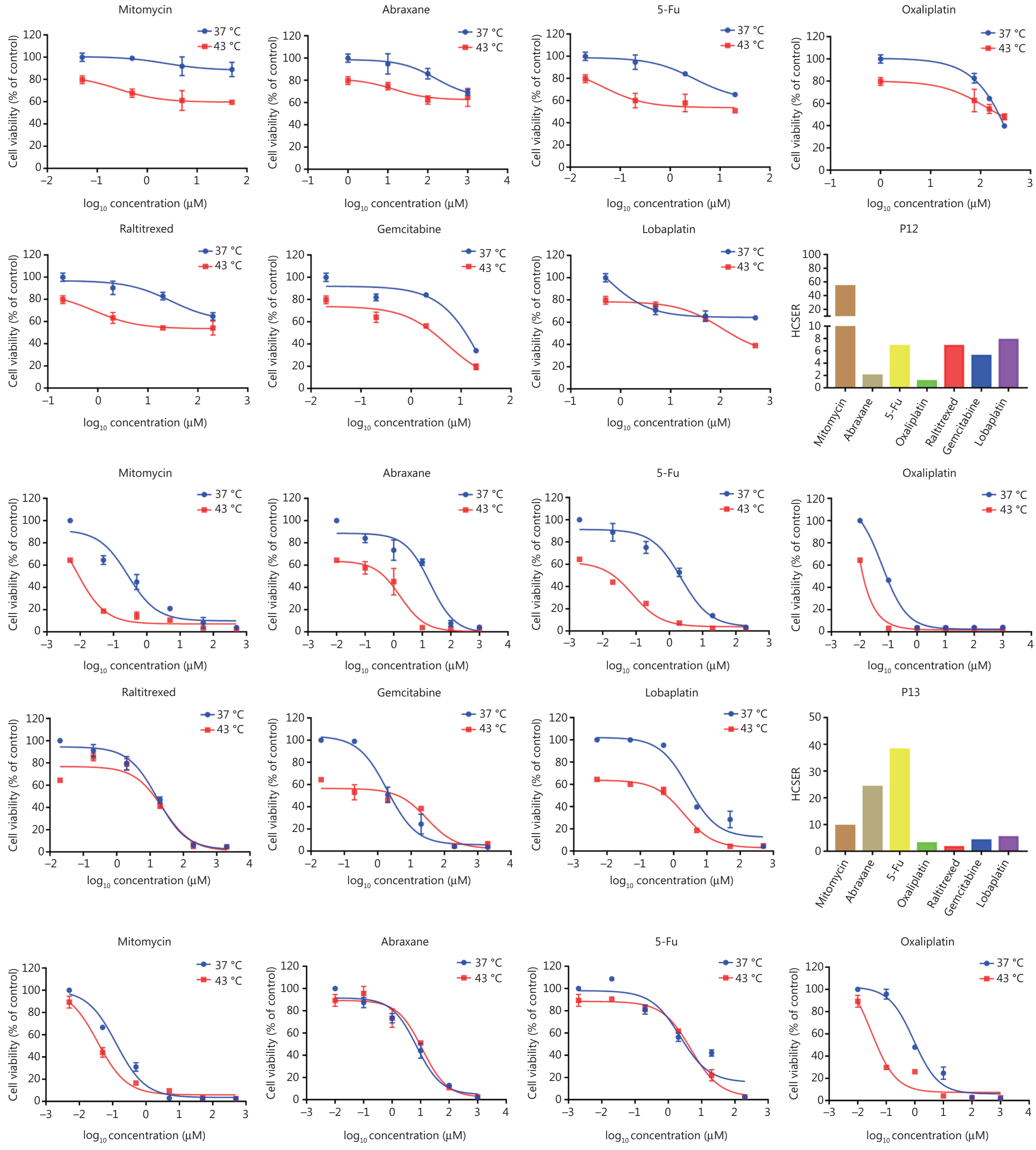

Figure S1 Continued

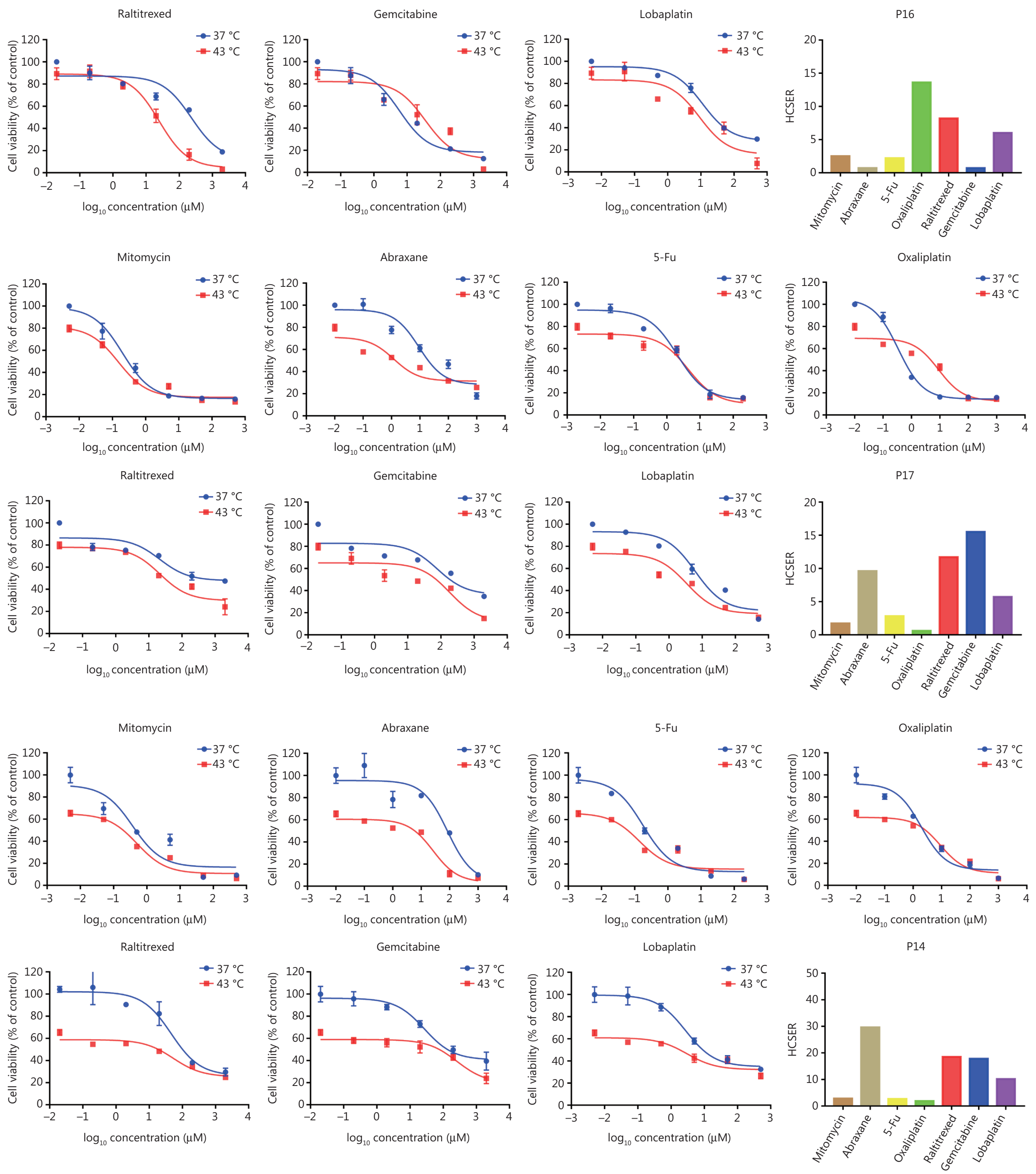

Figure S1 Continued

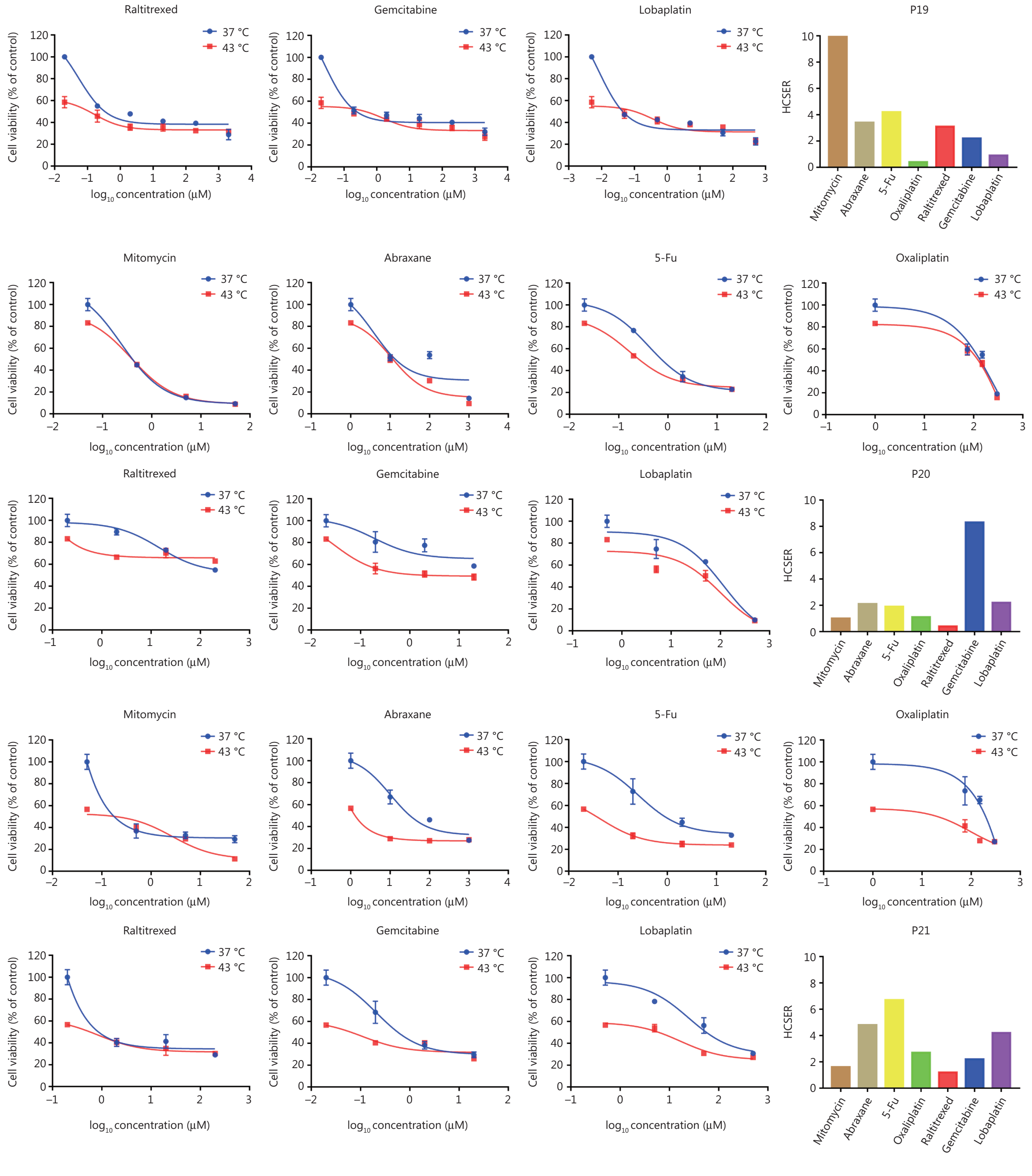

Figure S1 Continued

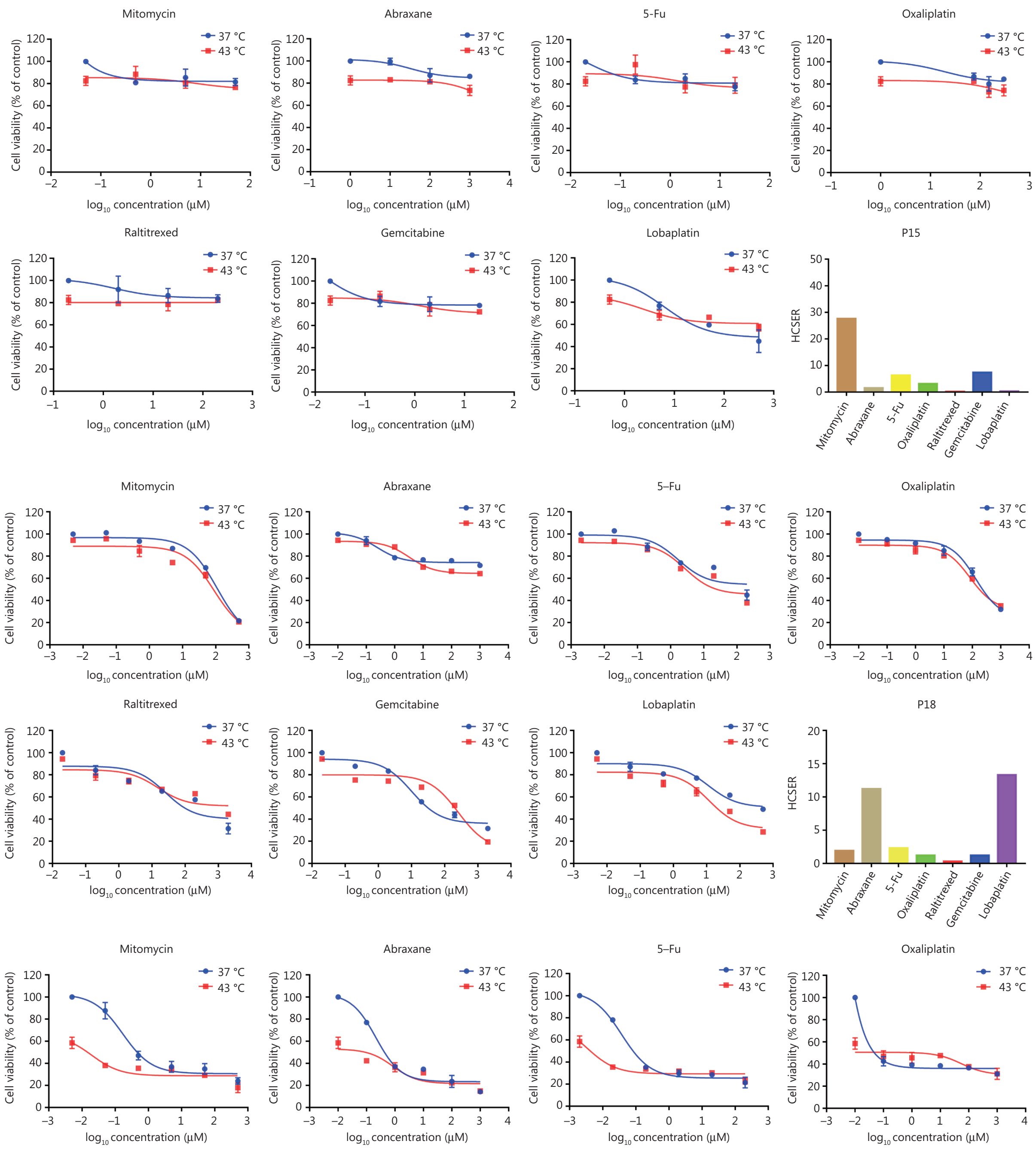

Figure S1 Continued

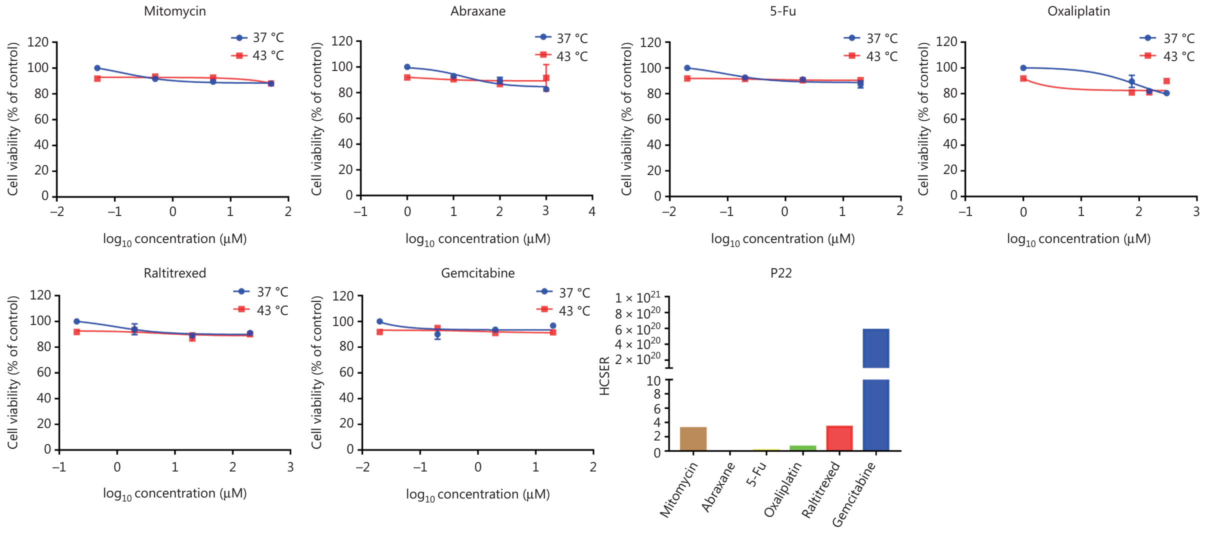

Figure S1 Drug response curves and hyperthermia chemotherapy sensitization enhancement ratio scores of organoids derived from the remnant patients in the 22-patient cohort.

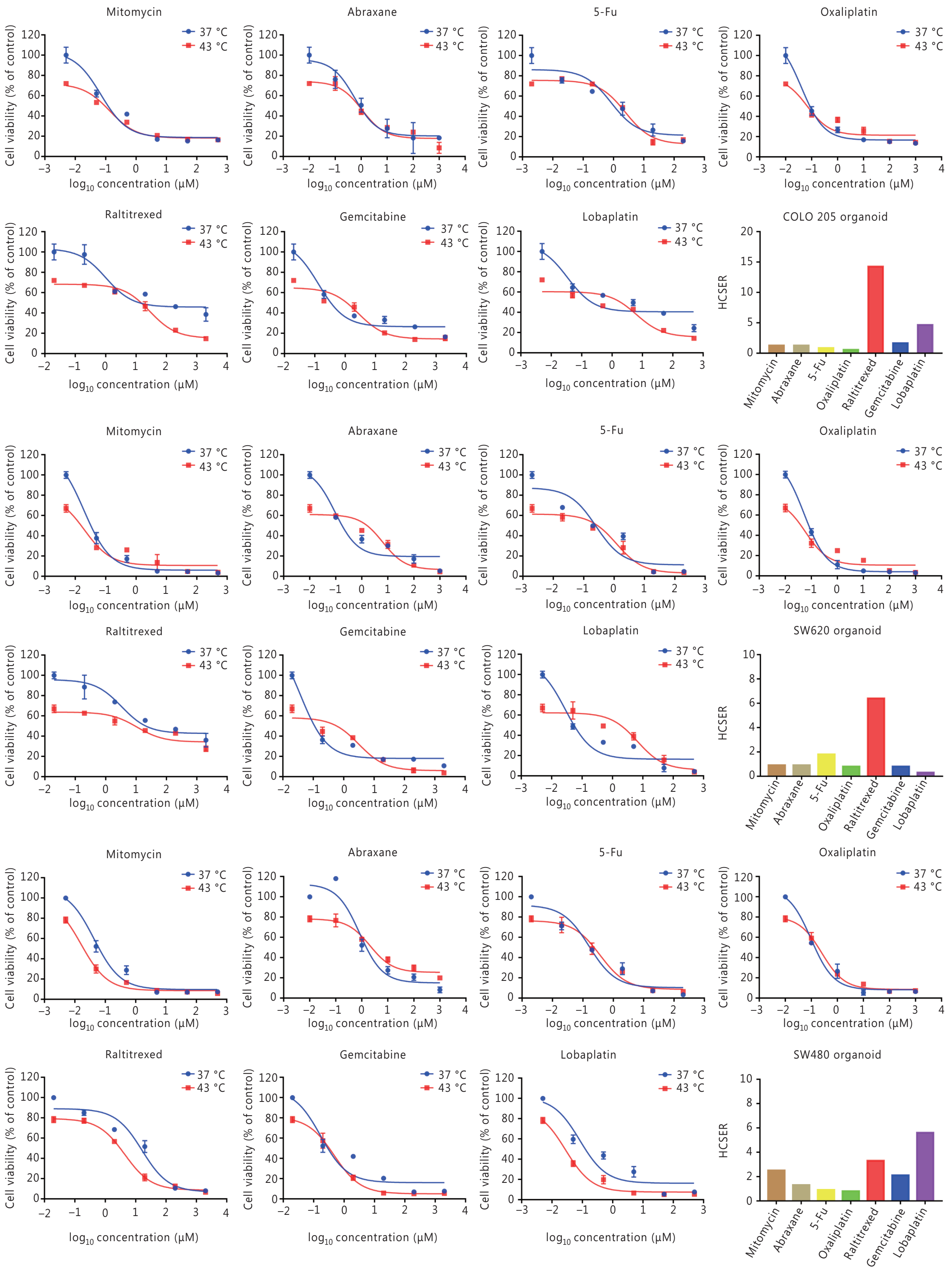

Figure S2 Drug response curves and hyperthermia chemotherapy sensitization enhancement ratio scores of organoids derived from colorectal cancer cell lines.

Table S1 Information on primary antibodies used in this study

Target	Supplier	Cat. No.	Application	Dilution
GAPDH	Sigma-Aldrich	G9545	WB	1:2,000
PI3K	ZEN BIO	350172	WB	1:1,000
Phospho-PI3K (Tyr607)	ZEN BIO	340790	WB	1:1,000
AKT	Cell Signaling Technology	4685	WB	1:1,000
Phospho-AKT (Thr308)	Cell Signaling Technology	13038	WB	1:1,000
4E-BP1	Cell Signaling Technology	9644	WB	1:1,000
Phospho-4E-BP1 (Thr37/46)	Cell Signaling Technology	2855	WB	1:1,000
Focal Adhesion Kinase	Affinity	AF6397	WB	1:500
Phospho-FAK (Tyr397)	Affinity	AF3398	WB	1:500

Table S2 All IC_{50} and standard deviation values of colorectal cancer patient samples and colorectal cancer cell lines

Drug	Average			Standard deviation		
	IC_{50} (37 °C)	IC_{50} (43 °C)	HCSER	37 °C	43 °C	HCSER
Patient 1						
Mitomycin	21.76	4.58	4.8	12.04	0.17	3.52
Abraxane	59.46	34.55	1.7	18.44	2.16	0.48
5-Fu	1.87	0.48	3.9	0.44	0.06	1.61
Oxaliplatin	16.08	4.28	3.8	1.79	0.50	0.85
Raltitrexed	19.14	1.9	10.1	4.40	0.26	4.56
Gemcitabine	42.41	32.03	1.3	6.17	5.41	0.42
Lobaplatin	0.77	0.37	2.1	0.10	0.03	0.63
Patient 2						
Mitomycin	72.81	0.75	97.1	7.18	0.06	36.46
Abraxane	64.86	14.76	4.4	21.75	0.01	2.41
5-Fu	63.46	4.30	14.8	8.61	2.72	6.87
Oxaliplatin	621.64	12.86	48.3	100.78	6.46	20.96
Raltitrexed	1,023.10	1.04	983.8	504.66	0.15	292.08
Gemcitabine	770.32	4.82	159.8	180.69	0.25	1.47
Lobaplatin	15.57	0.51	30.5	1.84	0.11	10.41
Patient 3						
Mitomycin	56.37	27.193	2.1	24.12	8.71	1.94
Abraxane	23,342.23	7,302.07	3.2	253.59	636.87	0.85
5-Fu	1,117.47	349.51	3.2	497.33	101.96	0.31
Oxaliplatin	1,470.59	1,185.52	1.2	480.41	227.88	1.21
Raltitrexed	1,875.68	5.865	319.8	178.95	0.35	84.66
Gemcitabine	58.72	23.41	2.5	41.90	6.57	1.24
Lobaplatin	17.37	5.361	3.2	12.46	2.47	4.54
Patient 4						
Mitomycin	1.58	0.73	2.2	0.20	0.01	0.47
Abraxane	1,325.14	1,682.37	0.8	675.00	199.87	0.27
5-Fu	3.47	1.09	3.2	1.06	0.32	2.75
Oxaliplatin	188.80	171.4	1.1	235.54	120.16	0.38
Raltitrexed	32,187.80	98.87	325.6	44,182.46	46.27	3.99
Gemcitabine	213.17	456.83	0.5	96.21	49.73	0.21
Lobaplatin	72.11	47.04	1.5	13.33	8.05	0.63
Patient 5						
Mitomycin	6.36	0.84	7.6	1.48	0.42	2.79
Abraxane	78.76	29.71	2.7	47.86	13.86	1.56

Table S2 Continued

Drug	Average			Standard deviation		
	IC_{50} (37 °C)	IC_{50} (43 °C)	HCSER	37 °C	43 °C	HCSER
5-Fu	2.98	0.33	9.0	0.45	0.15	7.97
Oxaliplatin	62.69	46.91	1.3	0.45	24.91	0.97
Raltitrexed	433.52	3.57	121.5	1,335.49	0.21	39.59
Gemcitabine	7.10	0.38	18.8	19.81	0.11	13.84
Lobaplatin	25.41	14.52	1.8	5.52	10.03	1.59
Patient 6						
Mitomycin	11.23	5.87	1.9	3.32	1.76	1.07
Abraxane	40.12	2.12	19.0	30.66	0.84	4.70
5-Fu	116.61	40.22	2.9	56.28	26.06	1.17
Oxaliplatin	1,565.39	463.15	3.4	187.35	143.96	0.88
Raltitrexed	15,755.51	462.76	34.0	8,008.95	137.89	6.27
Gemcitabine	1.51E+07	5.60E+05	27.0	3.38E+05	3.07E+03	3.76
Lobaplatin	918.38	3,041.62	0.3	230.62	7,221,353.16	0.01
Patient 7						
Mitomycin	41.81	3.55	11.8	1.86	0.37	1.42
Abraxane	518.38	349.15	1.5	59.90	87.15	0.20
5-Fu	22.4	10.21	2.2	8.33	1.84	1.30
Oxaliplatin	176.01	20.89	8.4	8.58	1.97	1.16
Raltitrexed	133.28	9.84	13.5	28.56	1.38	6.93
Gemcitabine	1,300.72	741.27	1.8	91.79	335.74	0.20
Lobaplatin	302.39	440.73	0.7	35.13	211.57	0.04
Patient 8						
Mitomycin	2.85	0.95	3.0	0.91	0.08	1.24
Abraxane	58.18	43.55	1.3	21.79	6.76	0.78
5-Fu	2.76	3.77	0.7	0.81	0.58	0.10
Oxaliplatin	59.87	25.73	2.3	16.18	3.01	0.53
Raltitrexed	235.60	18.81	12.5	39.32	2.17	4.30
Gemcitabine	138.04	118.16	1.2	47.63	9.24	0.31
Lobaplatin	41.04	29.02	1.4	8.09	6.62	0.04
Patient 9						
Mitomycin	0.24	0.09	2.7	0.07	0.02	1.16
Abraxane	1.08	2.00	0.5	0.39	0.47	0.21
5-Fu	0.10	0.25	0.4	0.03	0.04	0.26
Oxaliplatin	0.14	0.07	2.0	0.08	0.01	1.40

Table S2 Continued

Drug	Average			Standard deviation		
	IC_{50} (37 °C)	IC_{50} (43 °C)	HCSER	37 °C	43 °C	HCSER
Raltitrexed	11.73	1.67	7.0	2.76	0.36	3.86
Gemcitabine	0.25	0.47	0.5	0.06	0.10	0.24
Lobaplatin	0.48	1.10	0.4	0.07	0.15	0.14
Patient 10						
Mitomycin	0.89	0.70	1.3	0.02	0.06	0.77
Abraxane	79.02	36.28	2.2	10.77	3.92	1.75
5-Fu	1.29	0.80	1.6	0.20	0.05	0.77
Oxaliplatin	164.73	147.79	1.1	0.20	0.05	0.77
Raltitrexed	695.85	102.43	6.8	76.11	66.62	2.08
Gemcitabine	47.31	10.36	4.6	25.72	7.17	1.61
Lobaplatin	53.49	37.49	1.4	7.21	1.84	0.38
Patient 11						
Mitomycin	0.61	0.21	2.9	0.05	0.02	1.67
Abraxane	3.42	3.32	1.0	0.34	0.57	0.58
5-Fu	2.23	0.42	5.3	0.64	0.04	0.97
Oxaliplatin	0.16	0.14	1.1	0.02	0.03	0.89
Raltitrexed	27.49	4.26	6.5	0.38	2.44	1.12
Gemcitabine	2.87	0.74	3.9	0.34	0.08	0.48
Lobaplatin	23.69	6.23	3.8	4.33	2.97	2.41
Patient 12						
Mitomycin	3,011.47	56.00	53.8	2,325.76	47.15	46.75
Abraxane	4,142.60	2,075.05	2.0	2,273.25	689.07	1.56
5-Fu	56.47	8.26	6.8	14.14	16.97	2.88
Oxaliplatin	224.66	204.31	1.1	14.14	16.97	2.88
Raltitrexed	648.13	95.23	6.8	55.59	32.06	4.13
Gemcitabine	9.47	1.80	5.3	1.54	0.45	2.97
Lobaplatin	1,189.60	153.20	7.8	729.39	91.76	2.68
Patient 13						
Mitomycin	0.39	0.04	9.51	0.05	0.01	6.93
Abraxane	6.76	0.28	24.14	1.84	0.07	7.00
5-Fu	1.41	0.04	38.11	0.08	0.05	8.27
Oxaliplatin	0.12	0.04	2.93	0.003	0.01	0.38
Raltitrexed	12.26	7.61	1.61	1.18	1.48	0.91
Gemcitabine	3.42	0.83	4.12	1.33	0.13	1.87
Lobaplatin	4.91	0.94	5.22	0.97	0.003	2.57

Table S2 Continued

Drug	Average			Standard deviation		
	IC_{50} (37 °C)	IC_{50} (43 °C)	HCSER	37 °C	43 °C	HCSER
Patient 14						
Mitomycin	0.87	0.31	2.8	0.34	0.27	2.40
Abraxane	54.14	1.83	29.6	31.59	0.29	23.17
5-Fu	0.42	0.16	2.6	0.19	0.03	1.85
Oxaliplatin	3.27	1.68	1.9	1.56	0.25	0.75
Raltitrexed	157.21	8.48	18.5	23.55	0.62	13.68
Gemcitabine	344.73	19.35	17.8	311.73	11.21	12.55
Lobaplatin	33.78	3.31	10.2	16.08	1.64	3.60
Patient 15						
Mitomycin	27,299.88	989.63	27.6	6,545.77	512.34	23.35
Abraxane	27,890.85	18,267.79	1.5	19,434.59	9,312.66	0.01
5-Fu	1,207.98	191.96	6.3	528.39	319.43	2.26
Oxaliplatin	3,122.11	1,022.56	3.1	438.90	432.43	1.35
Raltitrexed	21,832.33	87,988.16	0.2	7,162.54	2,564.01	0.03
Gemcitabine	1,181.07	159.28	7.4	153.85	20.06	0.23
Lobaplatin	189.05	583.19	0.3	147.73	209.89	0.05
Patient 16						
Mitomycin	0.21	0.08	2.6	0.07	0.02	0.20
Abraxane	5.03	6.9	0.7	0.53	1.92	0.44
5-Fu	2.04	0.92	2.2	0.59	0.13	0.28
Oxaliplatin	1.7	0.13	13.1	0.37	0.02	6.30
Raltitrexed	136.54	16.73	8.2	28.18	6.37	4.46
Gemcitabine	13.91	19.67	0.7	5.54	6.16	0.37
Lobaplatin	42.42	7.08	6.0	5.21	1.54	1.55
Patient 17						
Mitomycin	0.78	0.45	1.7	0.27	0.04	1.92
Abraxane	39.75	4.12	9.6	3.25	0.52	2.89
5-Fu	3.22	1.14	2.8	0.61	0.19	0.96
Oxaliplatin	1.53	2.54	0.6	0.19	0.16	0.07
Raltitrexed	545.97	46.59	11.7	246.36	13.84	8.42
Gemcitabine	224.21	14.45	15.5	20.39	1.71	8.07
Lobaplatin	14.03	2.46	5.7	2.09	0.12	2.32

Table S2 Continued

Drug	Average			Standard deviation		
	IC_{50} (37 °C)	IC_{50} (43 °C)	HCSER	37 °C	43 °C	HCSER
Patient 18						
Mitomycin	95.89	51.03	1.9	11.61	3.12	0.28
Abraxane	33,496.08	3,001.73	11.2	2,996.02	2,470.23	2.17
5-Fu	90.63	39.56	2.3	35.79	0.65	0.69
Oxaliplatin	281.39	236.72	1.2	4.38	3.82	0.00
Raltitrexed	202.86	715.82	0.3	72.55	263.21	0.15
Gemcitabine	109.27	90.29	1.2	16.28	5.20	0.11
Lobaplatin	327.83	24.65	13.3	31.67	0.43	1.08
Patient 19						
Mitomycin	3.70	0.37	10.1	1.84	0.01	2.70
Abraxane	2.22	0.66	3.4	0.45	0.04	3.28
5-Fu	0.52	0.13	4.1	0.04	0.002	2.12
Oxaliplatin	1.96	4.48	0.4	0.21	0.31	0.29
Raltitrexed	9.68	3.10	3.1	5.03	5.64	1.82
Gemcitabine	11.54	5.16	2.2	2.77	0.29	1.27
Lobaplatin	0.88	0.98	0.9	0.40	0.05	0.40
Patient 20						
Mitomycin	0.75	0.77	1.0	0.27	0.72	0.20
Abraxane	52.80	24.64	2.1	17.09	4.77	0.22
5-Fu	1.49	0.80	1.9	0.26	0.14	0.54
Oxaliplatin	123.61	108.40	1.1	0.26	0.14	0.54
Raltitrexed	220.62	578.30	0.4	64.53	0.26	0.04
Gemcitabine	40.02	4.84	8.3	23.02	1.64	4.09
Lobaplatin	50.71	23.47	2.2	7.09	4.89	0.94
Patient 21						
Mitomycin	1.53	0.96	1.6	0.42	0.08	0.74
Abraxane	94.99	19.67	4.8	21.31	0.46	2.12
5-Fu	2.52	0.37	6.8	1.37	0.06	3.04
Oxaliplatin	177.32	66.74	2.7	32.49	23.48	1.37
Raltitrexed	8.76	7.46	1.2	3.17	0.59	0.93
Gemcitabine	1.68	0.77	2.2	0.59	0.73	2.11
Lobaplatin	91.08	21.9	4.2	49.99	3.14	1.49

Table S2 Continued

Drug	Average			Standard deviation		
	IC_{50} (37 °C)	IC_{50} (43 °C)	HCSER	37 °C	43 °C	HCSER
Patient 22						
Mitomycin	55,054.80	33,432.07	1.6	3,598.61	998.24	0.68
Abraxane	68,460.42	35,045,104	0.002	29,604.49	336,675.47	0.001
5-Fu	11,383.49	252,890.2	0.05	6,887.40	81,579.59	0.03
Oxaliplatin	2,351.46	9,320.58	0.3	1,887.40	8.16	0.05
Raltitrexed	647,384.3	377,677.7	1.7	19,458.87	20,062.92	1.25
Gemcitabine	3.47E+22	66,969.59	5.18E+22	1.02E+18	58,474.2835	3.28E+17
Lobaplatin	/	/	/	/	/	/
DLD-1						
Mitomycin	0.21	0.10	2.0	0.07	0.01	1.73
Abraxane	1.26	0.90	1.4	0.22	0.02	0.85
5-Fu	0.54	0.38	1.4	0.08	0.05	0.73
Oxaliplatin	0.24	0.28	0.9	0.06	0.04	0.45
Raltitrexed	103.22	6.62	15.6	17.32	0.34	5.94
Gemcitabine	0.93	0.35	2.7	0.12	0.04	1.36
Lobaplatin	0.39	0.11	3.7	0.10	0.001	2.26
COLO 205						
Mitomycin	0.45	0.31	1.4	0.14	0.05	1.18
Abraxane	2.51	1.76	1.4	1.33	0.64	1.33
5-Fu	1.41	1.34	1.1	0.59	0.52	0.80
Oxaliplatin	0.33	0.48	0.7	0.09	0.09	2.57
Raltitrexed	128.52	8.93	14.4	60.83	2.59	5.12
Gemcitabine	2.74	1.52	1.8	1.03	0.28	1.53
Lobaplatin	4.67	0.96	4.8	2.19	0.24	2.27
SW620						
Mitomycin	0.07	0.07	1.0	0.01	0.01	0.62
Abraxane	0.81	0.94	0.9	0.20	0.12	0.39
5-Fu	0.28	0.15	1.8	0.04	0.02	1.16
Oxaliplatin	0.13	0.17	0.8	0.02	0.02	1.41
Raltitrexed	125.20	19.46	6.4	70.71	4.02	2.53
Gemcitabine	0.56	0.68	0.8	0.13	0.11	0.63
Lobaplatin	0.23	0.79	0.3	0.07	0.17	0.10

Table S2 Continued

Drug	Average			Standard deviation		
	IC_{50} (37 °C)	IC_{50} (43 °C)	HCSER	37 °C	43 °C	HCSER
SW480						
Mitomycin	0.15	0.06	2.5	0.02	0.004	0.14
Abraxane	1.77	1.38	1.3	0.40	0.82	0.27
5-Fu	0.23	0.25	0.9	0.04	0.03	0.47
Oxaliplatin	0.28	0.35	0.8	0.06	0.02	0.63
Raltitrexed	11.23	3.39	3.3	5.64	0.97	2.43
Gemcitabine	1.07	0.51	2.1	0.36	0.11	1.22
Lobaplatin	0.39	0.07	5.6	0.10	0.002	3.71
